# Green Synthesis of Silver-Decorated Magnetic Particles for Efficient and Reusable Antimicrobial Activity

**DOI:** 10.3390/ma14247893

**Published:** 2021-12-20

**Authors:** Sachin V. Otari, Vipin Chandra Kalia, Aarti Bisht, In-Won Kim, Jung-Kul Lee

**Affiliations:** 1 Department of Chemical Engineering, Konkuk University, Seoul 05029, Korea; sachinotari169@gmail.com (S.V.O.); vckalia@gmail.com (V.C.K.); aartibisht94@gmail.com (A.B.); inwon@konkuk.ac.kr (I.-W.K.); 2 Institute of SK-KU Biomaterials, Konkuk University, Seoul 05029, Korea

**Keywords:** hybrid structure, green synthesis, antimicrobial activity

## Abstract

Metal and metal hybrid nanostructures have shown tremendous application in the biomedical and catalytic fields because of their plasmonic and catalytic properties. Here, a green and clean method was employed for the synthesis of silver nanoparticle (Ag NP)-SiO_2_-Fe_2_O_3_ hybrid microstructures, and biomolecules from green tea extracts were used for constructing the hybrid structures. The SiO_2_-Fe_2_O_3_ structures were synthesized using an ethanolic green tea leaf extract to form Bio-SiO_2_-Fe_2_O_3_ (BSiO_2_-Fe_2_O_3_) structures. Biochemical studies demonstrated the presence of green tea biomolecules in the BSiO_2_ layer. Reduction of the silver ions was performed by a BSiO_2_ layer to form Ag NPs of 5–10 nm in diameter in and on the BSiO_2_-Fe_2_O_3_ microstructure. The reduction process was observed within 600 s, which is faster than that reported elsewhere. The antimicrobial activity of the Ag-BSiO_2_-Fe_2_O_3_ hybrid structure was demonstrated against *Staphylococcus aureus* and *Escherichia coli*, and the nanostructures were further visualized using confocal laser scanning microscopy (CLSM). The magnetic properties of the Ag-BSiO_2_-Fe_2_O_3_ hybrid structure were used for studying reusable antimicrobial activity. Thus, in this study, we provide a novel green route for the construction of a biomolecule-entrapped SiO_2_-Fe_2_O_3_ structure and their use for the ultra-fast formation of Ag NPs to form antimicrobial active multifunctional hybrid structures.

## 1. Introduction

For the last seven decades since the discovery of antibiotics, their uncontrolled use has led to a generation of antibiotic-resistant strains that are unresponsive to currently available conventional antibiotics, creating a major concern for the health sector [1,2]. More new-generation antibiotics have been introduced in the field of medicine; however, the toxicity and adverse effects of these antibiotics are worrying factors for implementation [3,4]. Therefore, alternate therapeutic strategies have been studied for the last two decades, and several types of nanomaterials have been tested for their antimicrobial activity that is nontoxic to human and nonhuman hosts [5,6,7]. Silver nanoparticles (Ag NPs) have been extensively studied because of their effective antimicrobial activity known since ancient times [8]. A detailed study of the antimicrobial activity of Ag NPs against a wide range of pathogenic microorganisms was performed; it was demonstrated that the Ag NPs act by releasing Ag+ ions, which act on bacteria through reactive oxygen species [8]. The antimicrobial activity of the Ag NPs decreases with aggregate formation, where Ag NPs form a bulky material that makes it difficult for them to act on small bacteria and viruses [9,10,11]. In addition, Ag NPs are not only toxic to the bacteria but also to mammalian cells, which is a major concern for the therapeutic use of Ag NPs [12]. Therefore, several functionalizations or immobilization procedures have been used to enhance their antimicrobial efficiency and avoid the aggregation and toxicity of Ag NPs before release into the environment [13]. Currently, extensive research is ongoing for the construction of an ideal support to maintain the activity, stability, and reusability of Ag NPs [14].

Magnetic nanoparticles have been used as carriers for several nanomaterials, as they facilitate the separation of the nanoparticles by an external magnetic field and stability of the catalyst, which can reduce the adverse effects of the Ag NPs after use and reuse of Ag NPs for repeated applications [15]. Several researchers have demonstrated various methods for the synthesis of metal nanoparticle-supported SiO_2_ NPs and magnetic nanoparticles; these methods are time-consuming and require hazardous materials and expensive instruments with harsh reaction procedures [16,17]. Ag@nanosilica NPs have been synthesized using a protein extract of *Rhizopus oryzae* after 72 h of incubation [18]. Kaloti et al. used glucose to form Ag–γ-Fe_2_O_3_ nanocomposites by applying a very high temperature [19]. Tzounis et al. used polyethyleneimine for the functionalization of SiO_2_ nanospheres, wherein Ag NPs were decorated using sodium borohydride (NaBH_4_) as the reducing agent [20]. Muthukumar et al. prepared silver ferrite nanoparticles from *A. blitum* leaf extract at 50 °C and pH 9 after 4 h of reaction time [21]. To the best of our knowledge, this is the first report where reducing molecules, i.e., plant leaf extract immobilized in the silica layer of silica-coated magnetic nanoparticles, were used for the reduction of Ag^+^ ions to form metal-magnetic hybrid microstructures with ultrafine, spherical Ag NPs on the surface, within a short reaction duration and under mild reaction conditions. A major advantage of the hybrid structure synthesis method is the minimization of steps involving very complex, expensive, and cumbersome functionalization and reduction reaction conditions.

In this report, we present an economical and clean method for the construction of Ag-SiO_2_-Fe_2_O_3_ hybrid structures by entrapping biomolecules from green tea leaf extract of *Camellia sinensis*, thus forming BioSiO_2_ (BSiO_2_)-Fe_2_O_3_ particles. The entrapped biomolecules reduced Ag^+^ ions and formed Ag NPs on the BSiO_2_-Fe_2_O_3_ particles. Different techniques were used for analyzing the crystal structure and morphology of the prepared hybrid structures, such as X-ray diffraction (XRD), high-resolution transmission electron microscopy (HR-TEM), and magnetic property measurement using a superconducting quantum interference device (SQUID). The prepared hybrid structures showed reusable active antimicrobial activity.

## 2. Materials and Methods

### 2.1. Materials

Analytical reagent-grade (AR-grade) chemicals were used for all experiments without purification. Silver nitrate (AgNO_3_), iron (III) nitrate nonahydrate (Fe(NO_3_)_3_·9H_2_O), ethanol (C_2_H_5_OH; 99.9%), tetraethyl orthosilicate (TEOS), sodium carbonate, gallic acid monohydrate, 2,2-diphenyl-1-picrylhydrazyl (DPPH), 2,2′-azino-bis(3-ethylbenzothiazoline-6-sulphonic acid) (ABTS), potassium persulfate (K_2_Cr_2_O_7_), and ammonium hydroxide (NH_4_OH) were purchased from Sigma-Aldrich Co. Ltd. (St. Louis, MO, USA). Green tea bags (Jeju company, Korea) were purchased from the local market.

### 2.2. Preparation of the Ethanolic Extract of Green Tea Leaves

Green tea leaves (1.2 g) were ground and refluxed for 4 h in anhydrous ethyl alcohol (99.9%) under magnetic stirring. The suspension was cooled at room temperature and centrifuged at 4000 rpm for 10 min to remove large particulates of green tea leaves. Next, the green tea leaf extract was filtered through Whatman filter paper to remove small leaf particulates. Before further experimental procedures, the purified ethanolic green tea extract was kept in the dark at 4 °C.

### 2.3. Synthesis of Fe_2_O_3_ NPs

Fe_2_O_3_ NPs were synthesized using a previously reported method [22]. Briefly, 1 M of iron nitrate Fe(NO_3_)_3_ was prepared in 50 mL of distilled water (DW) under N_2_ gas bubbling for 30 min. The pH of this solution was maintained at more than 10 under vigorous stirring by using aqueous ammonium hydroxide (30%). Then, the solution was autoclaved at 150 °C for 10 h in a Teflon-lined stainless-steel reactor. The formed precipitate was centrifuged and washed several times with ethanol and water until a neutral pH was obtained. The particles were vacuum-dried at 50 °C for 12 h.

### 2.4. Synthesis of BSiO_2_-Fe_2_O_3_ Particles

BSiO_2_-Fe_2_O_3_ particles were synthesized using a previously reported method with modifications [23]. In the purified ethanolic green tea extract (40 mL), 50 mg of Fe_2_O_3_ NPs were dispersed under sonication for 20 min. Two milliliters of TEOS was added dropwise to the Fe_2_O_3_ NP suspension and further mixed with mechanical stirring for 1 h. The pH of the solution was adjusted to 11 by adding aqueous ammonium hydroxide dropwise. After 24 h of incubation, the formed particles were collected by centrifugation and repeatedly washed with ethanol and water. The BSiO_2_-Fe_2_O_3_ particles were vacuum-dried at 50 °C for 12 h.

### 2.5. Synthesis of the Ag-BSiO_2_-Fe_2_O_3_ Hybrid Microstructure

For the construction of the Ag-BSiO_2_-Fe_2_O_3_ hybrid structure, the BSiO_2_-Fe_2_O_3_ particles were dispersed in 10 mL of DW and sonicated for 20 min to disturb the agglomerates. Ten milliliters of aqueous AgNO_3_ was poured into the solution to form a 10 mM AgNO_3_ solution. The mixture was further incubated and stirred for 30 min and observed for color change. The formed hybrid structure was washed with water and ethanol several times, obtained with centrifugation, and vacuum-dried at 50 °C overnight.

### 2.6. Antimicrobial Activity

The antimicrobial activity of the Ag-BSiO_2_-Fe_2_O_3_ hybrid microstructure was tested against *Staphylococcus aureus* and *Escherichia coli*. The growth media used for *S. aureus* and *E. coli* were trypticase soy broth and Luria–Bertani broth, respectively. The bacterial cultures were grown in the broths for 8 h to obtain the desired optical density (OD). Initially, the effect of the hybrid structure, streptomycin, AgNPs, and Fe_2_O_3_ NPs was tested on the growth curve of the bacterial cells for 22 h. The growth of both microorganisms was monitored using UV-visible spectroscopy, that is, optical density at 610 nm. Various concentrations of the hybrid structure, from 50 to 90 µg mL^−1^, were added to the broths (10^6^ to 10^7^ bacterial cells mL^−1^) and further incubated for 24 h at 37 °C. The antimicrobial activity of streptomycin, AgNPs, and Fe_2_O_3_ NPs was also studied with varied concentrations. After incubation, 0.1 mL from the incubated broth was spread over the respective agar and incubated for 24 h and observed for growth, and the colony-forming units (CFUs) mL^−1^ were measured. The higher concentrations of the hybrid nanostructure inhibited the growth of the bacteria completely, and they were used for the antimicrobial reusability experiment, in which 200 µg mL^−1^ of the hybrid structure was inoculated in 10^6^ to 10^7^ bacterial cells mL^−1^. To visualize the antimicrobial activity of the prepared particles, a live/dead staining assay was performed after the exposure of 50 and 200 µg mL^−1^ of the nanohybrid nanostructure to *S. aureus* and *E. coli* cultures, as specified in earlier reports. The cells were observed under CLSM (Carl Zeiss LSM 710, Jena, Germany) [24].

### 2.7. Biochemical Analysis

#### 2.7.1. Estimation of Total Phenolic Content

The total phenolic content of the green tea extract prepared using water and ethanol was determined using the McDonald method [25]. Briefly, a standard solution of gallic acid monohydrate (100 μg mL^−1^) was prepared in methanol. Different concentrations of 0.1 to 1.0 mL of green tea leaf extract were mixed with a 1:1 mixture of Folin–Ciocalteu reagent and DW in 10 different tubes. To each tube, 7.5% sodium carbonate (2 mL) was added, and the mixture was allowed to react further for 30 min. The absorbance was measured at 765 nm, and the standard calibration curve was plotted. Then, 100 μL of the test samples was used to estimate the total phenolic content with a standard graph in terms of gallic acid equivalent (mg GAE g^−1^ extract).

#### 2.7.2. DPPH Radical Scavenging Activity

The antioxidant properties of the ethanolic green tea extract and BSiO_2_ NPs were measured based on the DPPH free radical scavenging activity [26]. In 100 mL of methanol, DPPH (6 mg) solution was prepared. The antioxidant activity was measured based on the discoloration of stable DPPH radicals in methanol, and at 517 nm, the absorbance was obtained. Different aliquots of the test samples were tested for the discoloration of DPPH solution. Using the given formula, the radical scavenging activity was calculated as follows: % Radical scavenging activity = absorbance (control) − absorbance (sample)/absorbance (control) × 100.

#### 2.7.3. ABTS Radical Scavenging Assay

The ABTS radical scavenging assay was followed to demonstrate the antioxidant capability of the ethanolic green tea extract before and after TEOS hydrolysis by using a previously published method with some modifications [26]. Briefly, green tea extract and solution obtained from TEOS hydrolysis were diluted to different concentrations. They were added to 7 mM of ABTS standard solution prepared with 2.45 mM of potassium persulfate (K_2_S_2_O_8_), and the mixture was further allowed to react for 6 min at 30 °C. After incubation, absorbance was measured at 734 nm. The radical scavenging activities of the extracts were calculated using the following equation and were compared with that of BHT: ABTS radical scavenging activity = absorbance (control) − absorbance (sample)/absorbance (control) × 100.

### 2.8. Analytical Instrumentation

UV-Visible spectroscopy was performed using a Schimadzu UV 1600 spectrophotometer with a 1 cm well-stoppered quartz cuvette. The morphology of the microstructures was evaluated by HR-TEM analysis using a JEM-3010 (JEOL) working at 300 kV and by energy-dispersive spectroscopy (EDS). Crystallography analysis of the nanostructures was performed using an X-ray diffractometer (Rigaku, Tokyo, Japan) with a Cu target. The magnetic properties of Fe_2_O_3_ and Fe_2_O_3_ hybrid structures were measured with a vibrating sample magnetometer (VSM; Lakeshore Cryotronics, Inc., Westerville, OH, USA). The antimicrobial activity was observed under CLSM. Dynamic light scattering (DLS) spectroscopy and zeta potential measurement of the particles in colloidal solution were performed using a NICOMP^TM^ 380 ZIS (Santa Barbara, CA, USA) for the determination of the hydrodynamic diameter (HDD) and charge on the particles.

## 3. Results and Discussion

### 3.1. Synthesis of the Hybrid Microstructure

**Figure 1:**
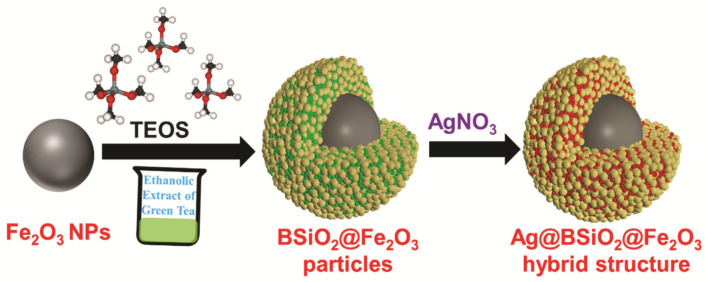
Schematics for the synthesis of silver-decorated biomolecule-entrapped SiO_2_-coated Fe_2_O_3_ (Ag-BSiO_2_-Fe_2_O_3_) hybrid structure.

The plant extract-mediated synthesis of nanomaterials has been one of the green chemistry approaches followed for the preparation of various materials. Extracts from several plant parts have been studied for the synthesis of metal nanoparticles that contain various polysaccharides, proteins, vitamins, or alkaloids, functionalizing and capping the nanomaterials with nontoxic and biodegradable biomaterials [27]. The presence of biomolecules on the surface of the nanoparticles leads to an increase in the colloidal stability of the nanoparticles forming a biomolecular corona on the surface, which contributes to enhanced bioactivity of the nanoparticles [28,29]. Here, in the presented work, the magnetic, catalytic active hybrid structure was synthesized with plant leaf extract biomolecules entrapped in SiO_2_ on the surface of the magnetic nanostructure and further dispersed in aqueous Ag^+^ ions for the reduction process to form Ag NPs on the BSiO_2_-Fe_2_O_3_ particle surface. Table S1 provides the total phenolic content of green tea in water and ethanol. Though the phenolic content in ethanol compared to water was low, ethanol is the most favorable for the extraction of total phenols because it prevents the oxidation of phenolic compounds. In addition, the antioxidant property of the ethanolic and water extract, obtained from ABTS and DPPH scavenging assays, revealed effective antioxidant activity in both solvents. In alkaline ethanolic green tea extract, TEOS was added for the formation of the SiO_2_ layer, where the biomolecules present in the green tea extract become trapped, resulting in biomolecule-entrapped SiO_2_ (BSiO_2_) (Figure 1).

**Figure 2:**
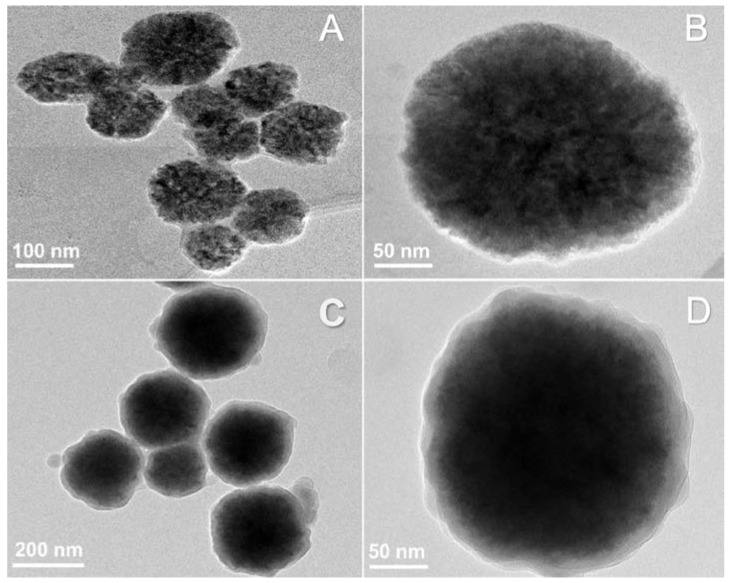
HR-TEM images of the (**A**,**B**) Fe_2_O_3_ nanosphere and (**C**,**D**) BSiO_2_-Fe_2_O_3_ particles.

The Fe_2_O_3_ NPs were synthesized using a previously reported hydrothermal method. Figure 2A,B show Fe_2_O_3_ NPs of an average diameter of 100 nm. To these magnetite NPs, the alkaline mixture of ethanolic green tea extract and TEOS was added and allowed to form the BSiO_2_ layer on the surface of the Fe_2_O_3_ NPs for 12 h at room temperature. In Figure 2C,D, the smooth layer of BSiO_2_ on the surface of the magnetite NPs can be observed to form a core–shell structure. The size of the magnetite NPs increased by approximately 30 nm, and a layer of 10–20 nm of BSiO_2_ was formed. The formed core–shell structure was exposed to aqueous AgNO_3_, and the active biomolecules of the BSiO_2_ layer reduced AgNO_3_ to form Ag NPs.

**Figure 3:**
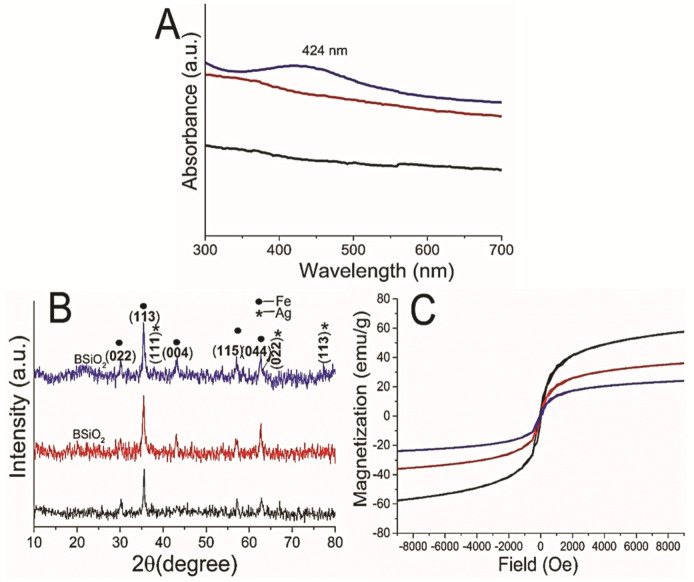
(**A**) UV-visible spectroscopic analysis of Fe_2_O_3_ NPs, BSiO_2_-Fe_2_O_3_ particles, and Ag-BSiO_2_-Fe_2_O_3_ structure. (**B**) XRD analysis. (**C**) Magnetization curves of γ-Fe_2_O_3_ NPs (black), BSiO_2_-Fe_2_O_3_ particles (red), and Ag- BSiO_2_-Fe_2_O_3_ structure (blue).

The synthesis of Ag NPs was confirmed using UV-visible spectroscopy (Figure 3A). No absorbance for Fe_2_O_3_ and BSiO_2_-Fe_2_O_3_ NPs was observed in the range of 400–500 nm, whereas Ag-BSiO_2_-Fe_2_O_3_ NPs showed characteristic absorbance at 424 nm, confirming the formation of Ag NPs on the BSiO_2_-Fe_2_O_3_ hybrid nanostructure. There are several reports on the synthesis of Ag NPs using biomaterials originating from plants and bacteria such as carbohydrates, amino acids, enzymes, proteins, and polyphenols [30]. Moulton et al. proposed a mechanism for the synthesis of Ag NPs using epigallocatechin-3-gallate obtained from *C. sinensis* leaf extract [31]. In addition, tea polyphenols, flavonoids, and epicatechin present in *C. sinensis* leaf extract were found to function as reducing and capping agents in the Ag NP synthesis process [31]. DFT calculations showed that the catechol moiety of flavonoids has a low energy of dissociation for the O–H bond of -OH groups, which is less than that of the -OH groups present in other parts of flavonoids [32]. The -OH groups of the catechol moiety of flavonoids have been demonstrated to function as metal-ion-reducing molecules. Various solvents have been utilized for the extraction of polyphenols and epicatechins from various plant extracts to maintain their high antioxidant activity [33]. Green tea extracts prepared in ethanol have high polyphenol content and retain high antioxidant properties [33]. Here, microhybrid structures were fabricated using the concentrated catechin derivatives from ethanolic green tea extract with active reducing capacity.

XRD spectra of the γ-Fe_2_O_3_ NPs (black), BSiO_2_-Fe_2_O_3_ particles (red), and Ag-BSiO_2_-Fe_2_O_3_ hybrid (blue) are shown in Figure 3B, demonstrating the crystallinity of the hybrid materials. The γ-Fe_2_O_3_ NPs showed a typical XRD pattern with the face-centered cubic structure of magnetite consistent with JCPDS card no. 96-101-3050 (Figure 3B, black). The characteristic peaks obtained at 2θ were 30.06°, 35.4°, 43.0°, 56.9°, and 62.5° and corresponded to (022), (113), (004), (115), and (044) indices, respectively [34]. The XRD data for BSiO_2_-Fe_2_O_3_ particles (Figure 3B, red) were similar to those for Fe_2_O_3_ NPs, with a broad peak of 21° at 2θ corresponding to the amorphous BSiO_2_ layer [20]. The XRD pattern of the Ag-BSiO_2_-Fe_2_O_3_ hybrid (Figure 3B, blue) showed characteristic peaks for Fe_2_O_3_ along with characteristic peaks for Ag of 37.8°, 63.9°, and 76.7° at 2θ corresponding to phase indices (111), (022), and (113), respectively (JCPDS card no. 969013048) [35]. The XRD pattern demonstrated the face-centered-cubic structure of metallic silver. A broad peak of 21–23° at 2θ for SiO_2_ was observed in the XRD of the Ag-BSiO_2_-Fe_2_O_3_ hybrid structure. The VSM measurement with a peak field of 20 KOe at room temperature was used to calculate the magnetic hysteresis loops of γ-Fe_2_O_3_ NPs (black), BSiO_2_-Fe_2_O_3_ NPs (red), and Ag BSiO_2_-Fe_2_O_3_ hybrid and to analyze changes in the magnetic behavior of the NPs with modifications (Figure 3C, blue). The saturation magnetization (M_s_) value of the γ-Fe_2_O_3_ NPs was 61 emu g^−1^ at 300 K. After modification with BSiO_2_ on the surface of the γ-Fe_2_O_3_ NPs, the saturation magnetization value reduced to 39 emu g^−1^, and it further declined to 23 emu/g because of the presence of Ag NPs in the BSiO_2_ layer. The decrease in the saturation magnetization may be due to the mass replacement and shielding of the magnetic NPs with SiO_2_ and Ag NPs. The remanent magnetization (Mr) and coercivity (Hc) of the bare Fe_2_O_3_ were also affected by the modifications with SiO_2_ and Ag NPs. The Mr decreased from 16.3 emu g^−1^ for γ-Fe_2_O_3_ NPs to 11.5 emu g^−1^ for BSiO_2_-Fe_2_O_3_ NPs and then to 6.4 emu g^−1^ for the Ag-BSiO_2_-Fe_2_O_3_ hybrid structure. The Hc value of Fe_2_O_3_ NP increased from 114 Oe to 121 Oe for the Ag-BSiO_2_-Fe_2_O_3_ hybrid structure. The increase in Hc may be attributed to changes in the domain structure or surface coating anisotropy. Although the Ag-BSiO_2_-Fe_2_O_3_ structure showed decreased saturation magnetization due to the presence of Ag NPs and SiO_2_, it displayed extreme magnetic behavior, which suggests its magnetic separation capability. Thus, the Ag-BSiO_2_-Fe_2_O_3_ nanohybrid can be recovered and reused by applying an external magnetic field. Therefore, the proposed method for modifying the SiO_2_ nanostructures can be used for the construction of various hybrid nanostructures.

**Figure 4:**
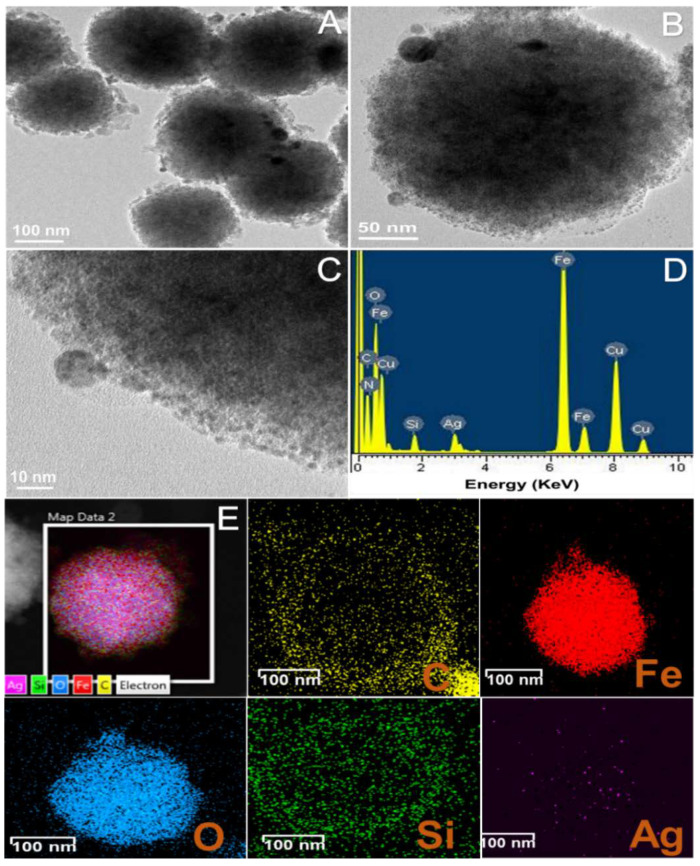
(**A**–**C**) HR-TEM images, (**D**) EDS spectrum analysis, and (**E**) elemental dot mapping of Ag-BSiO_2_-Fe_2_O_3_ hybrid structure.

Figure 4A,B show HR-TEM images of the hybrid structure demonstrating the presence of small Ag NPs on the surface of the BSiO_2_-Fe_2_O_3_ NPs, thus forming the Ag-BsiO_2_-Fe_2_O_3_ hybrid structure. The Ag NPs were 5–10 nm in size and were distributed evenly in and on the BsiO_2_ layer (Figure 4C). EDS analysis confirmed the presence of elemental silver in the hybrid nanostructure (Figure 4D). Figure 4E shows the elemental distribution of the Ag-BSiO_2_-Fe_2_O_3_ structure displaying the elemental maps of C, Fe, O, Si, and Ag individually. The elemental mapping clearly shows that biomolecules from the green tea extracts were well distributed in the SiO_2_ layer around the Fe_2_O_3_ nanoparticles, which further led to the synthesis of Ag NPs to form the hybrid nanostructure. The FT-IR spectrum of the extract of green tea and Ag-BSiO_2_-Fe_2_O_3_ NPs with the spectral range from 400 to 4000 cm^–1^ showed the presence of bands at ~3327 cm^−1^, ~2974 cm^−1^, and 2880 cm^−1^, corresponding to aliphatic OH groups, νCH_2_, and C-H stretching, confirming the presence of phenolic compounds in green tea and Ag-BSiO_2_-Fe_2_O_3_ NPs (Figure S1) [36]. The band at 1252 cm^−1^ corresponds to the vibrations of the C–O groups, which suggest the existence of polyols such as hydroxy flavonoids. The bands at 1044 cm^−1^ and 877 cm^−1^ correspond to CO stretching vibrations and C–C stretching vibrations, respectively, corresponding to the extract’s flavonoids. The spectra of Ag-BSiO_2_-Fe_2_O_3_ NPs at ~1074 cm^−1^ showed Si–O–Si stretching vibrations, confirming the presence of a SiO_2_ layer on the Fe_2_O_3_ NPs [37].

### 3.2. Antimicrobial Activity

**Figure 5:**
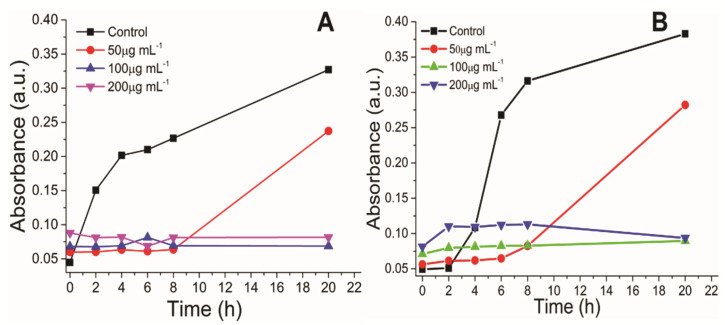
Growth curves of (**A**) *S. aureus* and (**B**) *E. coli* with various concentrations of the Ag-BSiO_2_-Fe_2_O_3_ hybrid structure.

**Figure 6:**
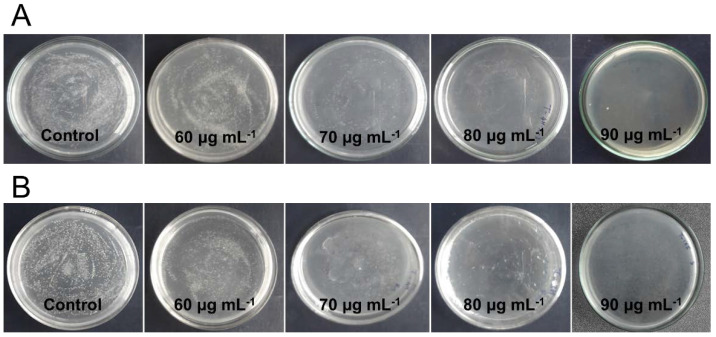
Growth plate photographs of (**A**) *S. aureus* and (**B**) *E. coli* with various concentrations of Ag-BSiO_2_-Fe_2_O_3_ hybrid structure.

The effective antimicrobial activity of the NPs is dependent on the NPs aggregation and interaction of the NPs with microbial cells. Thus, the hydrodynamic diameter (HDD) and zeta potential studies of the NPs provide information regarding the size and charge on the NPs in the colloidal solution, demonstrating the stability of NPs. The average HDD of the AgNPs, Fe_2_O_3_ NPs, and Ag-BSiO_2_-Fe_2_O_3_ hybrid structure in the saline solution was 30 (Figure S2A), 200 (Figure S2B), and 500 nm (Figure S2C), respectively. The zeta potential of the AgNPs, Fe_2_O_3_ NPs, and Ag-BSiO_2_-Fe_2_O_3_ hybrid structure was −28.6 (Figure S3A), −5.36 (Figure S3B), and −17.8 mV (Figure S3C), respectively. The antimicrobial activity of the streptomycin, AgNPs, Fe_2_O_3_ NPs, and Ag-BSiO_2_-Fe_2_O_3_ hybrid structure was analyzed using *S. aureus* (Gram-positive) and *E. coli* (Gram-negative). The effective concentration of the various structures was determined by analyzing the inhibitory effect on the growth curve of both microorganisms; the time-dependent growth of the microorganisms was monitored at 610 nm. Figure 5 shows that 50 µg mL^−1^ of the microhybrid affected the growth rates of *S. aureus* (Figure 5A) and *E. coli* (Figure 5B), but it did not completely inhibit both microorganisms. At 100 and 200 µg mL^−1^, the growth of both microorganisms was hampered, and no changes in the absorbance were observed after 20 h of incubation with the Ag-BSiO_2_-Fe_2_O_3_ structure. In similar experiments, a 10 µg mL^−1^ concentration of streptomycin and AgNPs showed an inhibitory effect on both microorganisms. The complete inhibition of *S. aureus* (Figure S4A) and *E. coli* (Figure S4B) was observed at 30 µg mL^−1^ of streptomycin, whereas 50 µg mL^−1^ of AgNPs showed complete inhibition of *S. aureus* (Figure S5A) and *E. coli* (Figure S5B). The Fe_2_O_3_ and BSiO_2_-Fe_2_O_3_ NPs showed no effect on the bacterial growth curve in 50–200 µg mL^−1^ for both microorganisms (Figures S6 and S7). It is clear that 50 µg mL^−1^ of the hybrid structure was most effective for *S. aureus*, whereas *E. coli* showed some inhibition at this concentration. However, both microorganisms were highly sensitive to 200 µg mL^−1^ of the Ag-BSiO_2_-Fe_2_O_3_ hybrid structure. To determine the minimum bactericidal concentration, both bacterial strains were exposed to various concentrations of the Ag-BSiO_2_-Fe_2_O_3_ structure. Although 50 µg mL^−1^ of the hybrid structure reduced the growth of the microorganisms, it did not inhibit growth completely. However, as the concentration of the microhybrid increased from 50 to 90 µg mL^−1^, the growth of both microorganisms constantly decreased. At 90 µg mL^−1^ of the hybrid structure, complete inhibition was observed for *S. aureus* (Figure 6A) and *E. coli* (Figure 6B); therefore, it can be considered the minimum bactericidal concentration (MBC) for both bacterial strains. For streptomycin, 20 µg mL^−1^ and 30 µg mL^−1^ were inhibitory for *S. aureus* and *E. coli*, respectively, as no growth was observed on growth plates (Figure S8). AgNPs inhibited both microorganisms at 30 µg mL^−1^ (Figure S9). However, the Fe_2_O_3_ and BSiO_2_-Fe_2_O_3_ particles showed no effect on the microbial growth in 50 to 90 µg mL^−1^ (Figures S10 and S11). The AgNPs showed high stability and low HDD in the aqueous solution, which allowed the maximum interaction with microorganisms, leading to its effective antimicrobial activity, whereas the presence of plant extract and AgNPs on the surface Fe_2_O_3_ NPs increased the stability of the hybrid nanostructure compared to Fe_2_O_3_ NPs, allowing it to perform more effectively in antimicrobial activity. The CLSM study was performed to visualize the antimicrobial activity of the hybrid structure where a 200 μg mL^−1^ concentration of the hybrid structure is completely inhibitory to both species showing the red dead bacteria cells (Figures S12 and S13).

**Figure 7:**
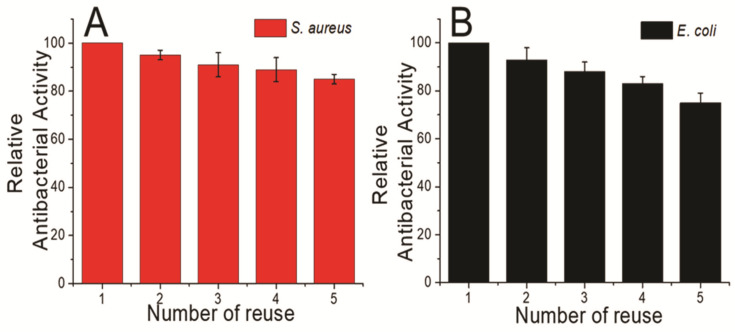
Reusable antibacterial effects of Ag-BSiO_2_-Fe_2_O_3_ hybrid structure (200 μg mL^−1^) on (**A**) *S. aureus* and (**B**) *E. coli*.

Because 200 µg mL^−1^ of the microhybrid caused complete inhibition of both pathogenic strains, the concentration of the hybrid structure was checked for repeated use of antimicrobial activity. The Ag-BSiO_2_-Fe_2_O_3_ hybrid was added to each bacterial suspension, and the mixture was incubated for 24 h. After incubation, the hybrid material was isolated with an external magnet, and the bacterial suspension was analyzed spectroscopically. The hybrid structure was washed with a buffer before each repeated use, and the new bacterial suspension was added to this washed buffer. This was repeated for the next 5 cycles. The Ag-BSiO_2_-Fe_2_O_3_ hybrid structure maintained 80% of its relative antimicrobial activity against *S. aureus* and 75% against *E. coli* (Figure 7). The loss of antimicrobial activity of the Ag-BSiO_2_-Fe_2_O_3_ hybrid structure may be due to the loss of the hybrid material during isolation from the bacterial suspension.

## 4. Conclusions

Here, we have developed a prominent, clean, robust method for the synthesis of an antimicrobial hybrid nanostructure using green tea active biomolecules. *C. sinensis* green tea ethanolic extract comprises polyphenols and other active ingredients and has a high antioxidant and reducing activity. The extract was allowed to become trapped in the SiO_2_ layer by hydrolysis. This process of entrapment was employed to synthesize the separable SiO_2_-coated Fe_2_O_3_ NPs magnetically. The reducing capacity of biomolecule-entrapped SiO_2_-Fe_2_O_3_ NPs was used to synthesize Ag NPs on the surface of the magnetic structure, thus forming the Ag-BSiO_2_-Fe_2_O_3_ hybrid nanostructure. As the entrapped biomolecules have a high reducing capacity, the reduction of Ag^+^ ions occurred quickly, which led to the formation of spherical Ag NPs with diameters in the range of 5–10 nm on the magnetic nanostructure. Elemental dot mapping revealed the uniform distribution of biomolecules and Ag NPs in the SiO_2_ layer coated on Fe_2_O_3_ NPs. The Ag-BSiO_2_-Fe_2_O_3_ hybrid microstructure showed excellent antimicrobial activity against *S. aureus* and *E. coli*. As the hybrid nanostructure was magnetic in nature, it showed excellent reusable antimicrobial activity. Toxicology studies need to be performed to use the antioxidant hybrid structure for medicinal and environmental applications. The outcome of the presented work creates opportunities for the construction of various metal microhybrid structures using eco-friendly biomaterials.

## Data Availability

Not applicable.

## Supplementary Materials

The following are available online at https://www.mdpi.com/article/10.3390/ma14247893/s1, Figure S1: FT-IR spectroscopy analysis of green tea extract and Ag-BSiO_2_ NPs-Fe_2_O_3_ NPs; Figure, S2: The Dynamic light scattering (DLS) analysis of colloids suspension of (A) Ag NPs, (B) Fe_2_O_3_ NPs, and (C) Ag-BSiO_2_- Fe_2_O_3_ NPs; Figure S3: The zeta potential analysis of colloids suspension of (A) Ag NPs, (B) Fe_2_O_3_ NPs, and (C) Ag-BSiO_2_- Fe_2_O_3_ NPs; Figure S4: Growth curves of (A) *S. aureus* and (B) *E. coli* with various concentrations of the streptomycin antibiotics; Figure S5: Growth curves of (A) *S. aureus* and (B) *E. coli* with various concentrations of the AgNPs; Figure S6: Growth curves of (A) *S. aureus* and (B) *E. coli* with various concentrations of the Fe_2_O_3_ NPs; Figure S7: Growth curves of (A) *S. aureus* and (B) *E. coli* with various concentrations of the BSiO_2_-Fe_2_O_3_ NPs; Figure S8: Growth plate photographs of (A) *S. aureus* and (B) *E. coli* with various concentrations of streptomycin antibiotics; Figure S9: Growth plate photographs of (A) *S. aureus* and (B) *E. coli* with various concentrations of Ag NPs; Figure S10: Growth plate photographs of (A) *S. aureus* and (B) *E. coli* with various concentrations of Fe_2_O_3_ NPs; Figure S11: Growth plate photographs of (A) *S. aureus* and (B) *E. coli* with various concentrations of BSiO_2_-Fe_2_O_3_ NPs; Figure S12: CLSM microscopy images of the antimicrobial activity of Ag-BSiO_2_-Fe_3_O_4_ nanoparticles against *E. coli*.; Figure S13: CLSM microscopy images of the antimicrobial activity of Ag-BSiO_2_-Fe_3_O_4_ nanoparticles against *S. aureus*. Table S1: Total phenolic contents and antioxidant activity of the green tea extract in different solvents.
