# Supplementary material for: Green Synthesis of Silver-Decorated Magnetic Particles for Efficient and Reusable Antimicrobial Activity
Source: Materials (Basel). 2021 Dec 20;14(24):7893. doi: 10.3390/ma14247893 (PMC8709440; doi:10.3390/ma14247893)
Supplement: Supplementary file 1 [file materials-14-07893-s001.zip › materials-1468564-supplementary.pdf]

Table S1. Total phenolic contents and antioxidant activity of the green tea extract in different solvents.

Sol-vents	Total Phenolics (μg of gallic acid equivalent mL^{-1} extract)	ABTS (μg of ascorbic acid equivalent mL^{-1} extract)	DPPH Activity (μg of ascorbic acid equivalent mL^{-1} extract)
Water	1346	2684	1869
Ethanol	104.6	342.6	173.8

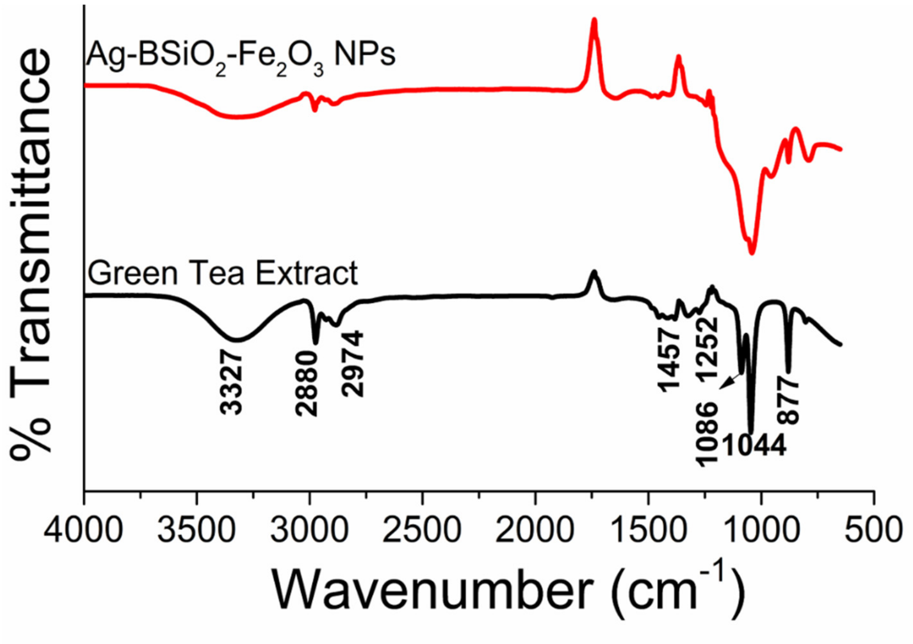

Figure S1. FT-IR spectroscopy analysis of green tea extract and Ag-$BSiO_2$ NPs-Fe_2O_3 NPs.

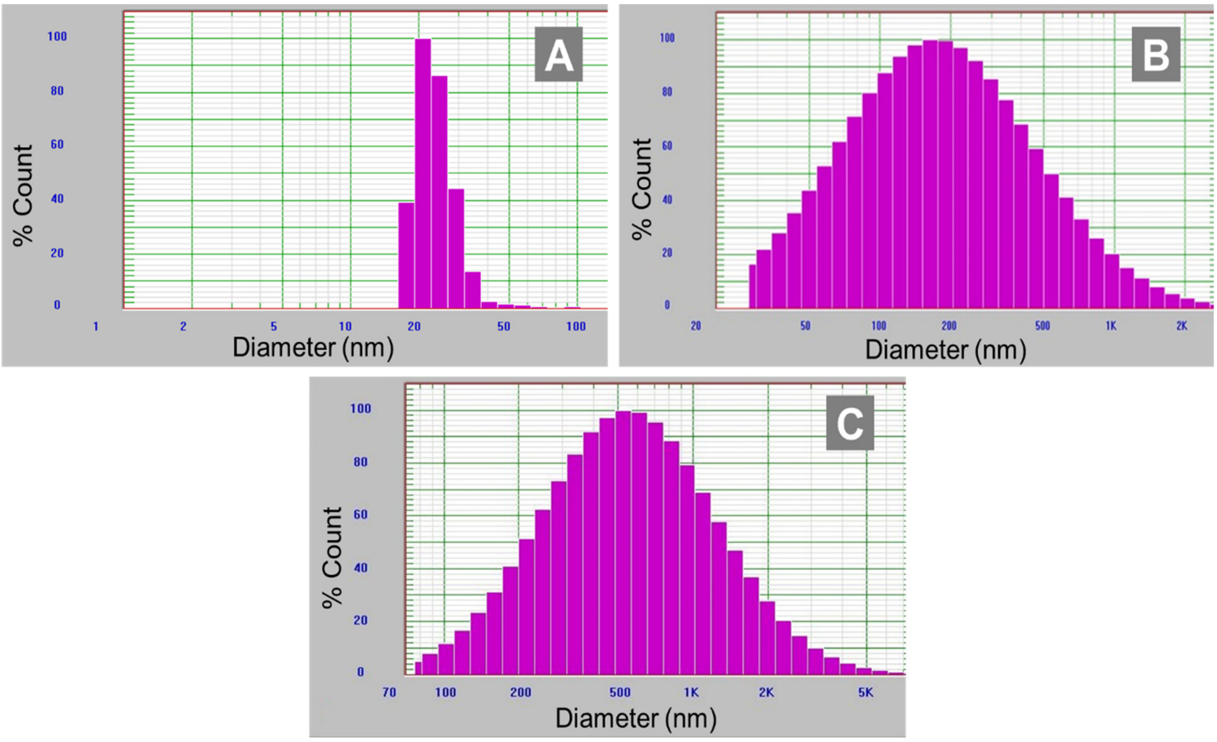

Figure S2. The Dynamic light scattering (DLS) analysis of colloids suspension of (**A**) Ag NPs, (**B**) Fe_2O_3 NPs, and (**C**) Ag-$BSiO_2$- Fe_2O_3 NPs.

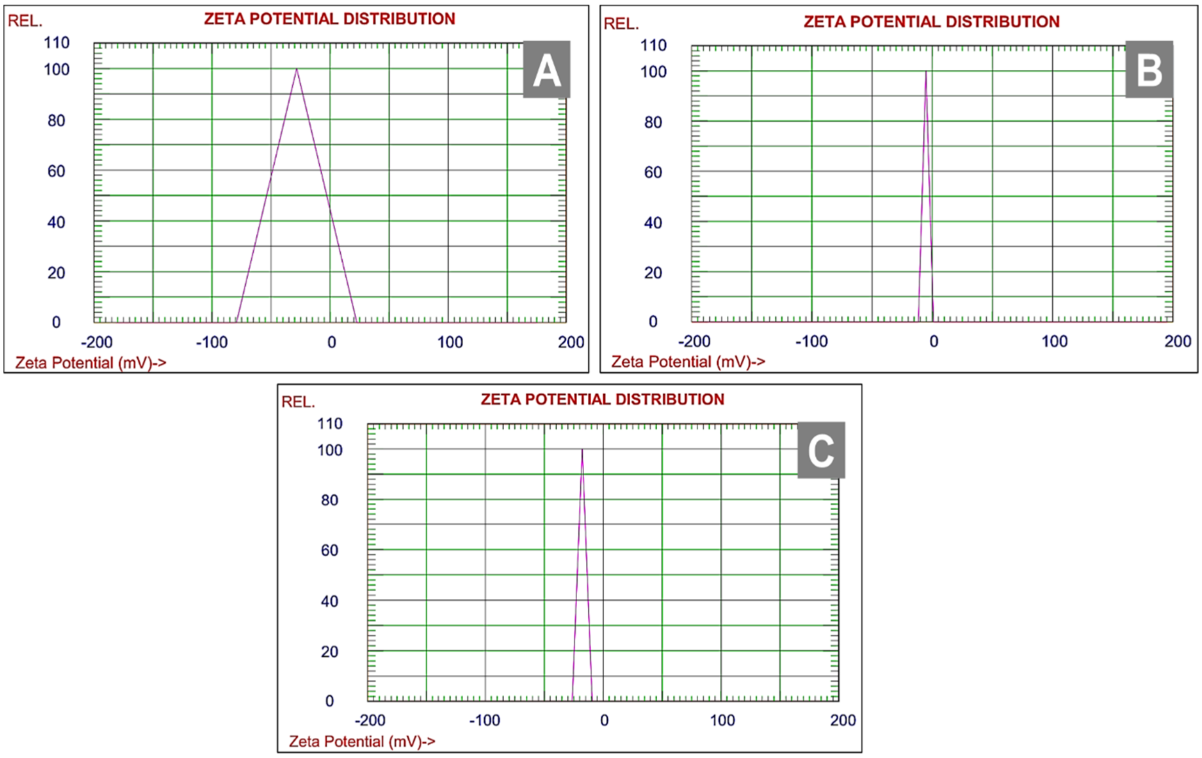

Figure S3. The zeta potential analysis of colloids suspension of (**A**) Ag NPs, (**B**) Fe_2O_3 NPs, and (**C**) Ag-BSiO$_2$- Fe_2O_3 NPs.

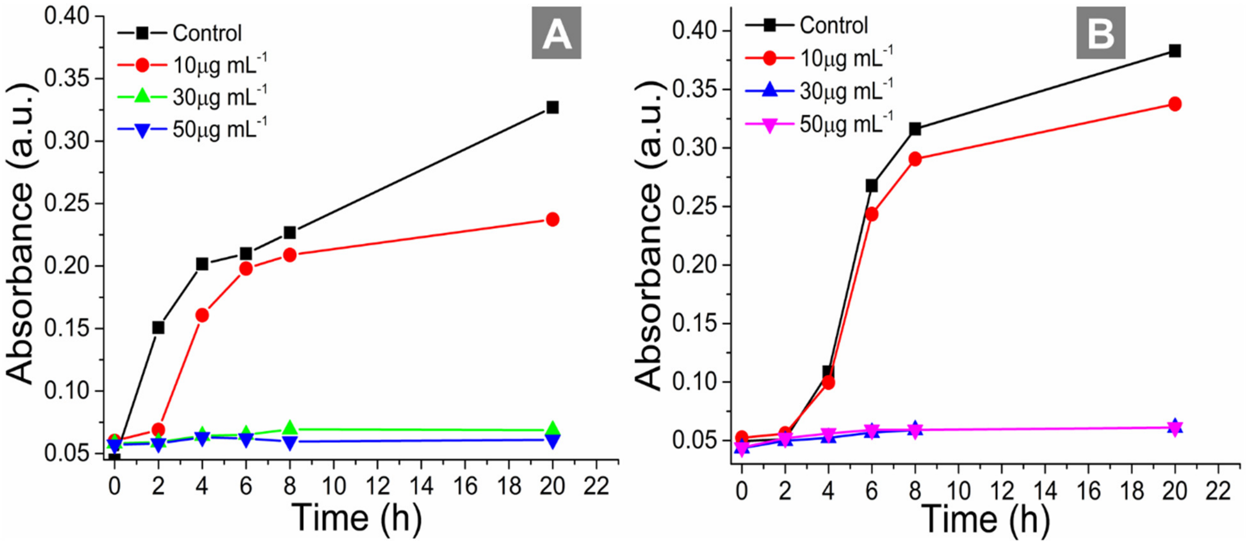

Figure S4. Growth curves of (**A**) *S. aureus* and (**B**) *E. coli* with various concentrations of the streptomycin antibiotics.

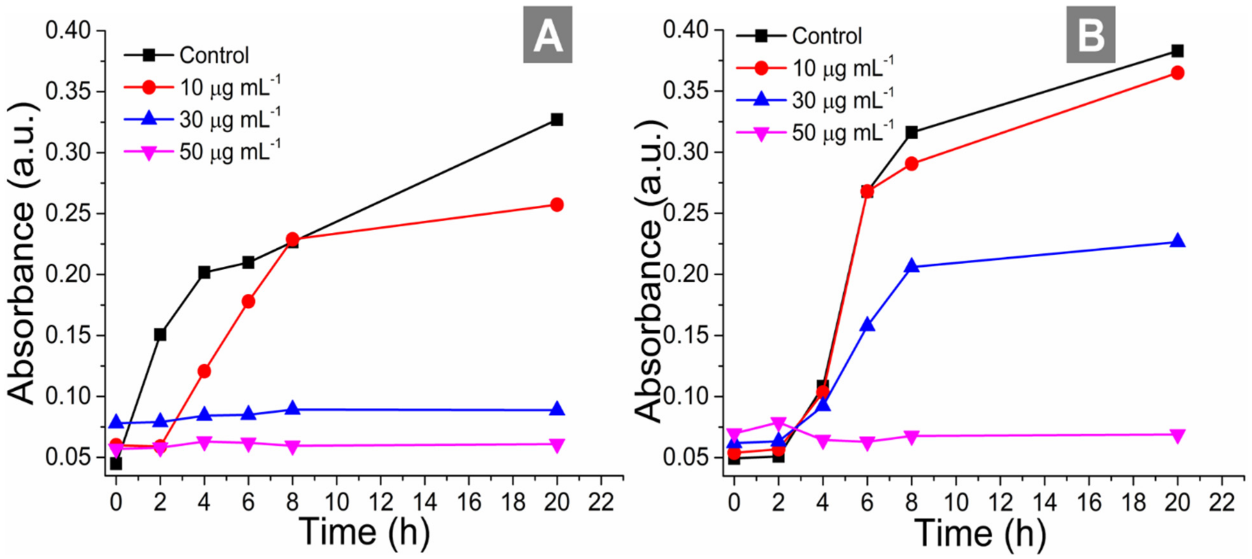

Figure S5. Growth curves of (**A**) *S. aureus* and (**B**) *E. coli* with various concentrations of the AgNPs.

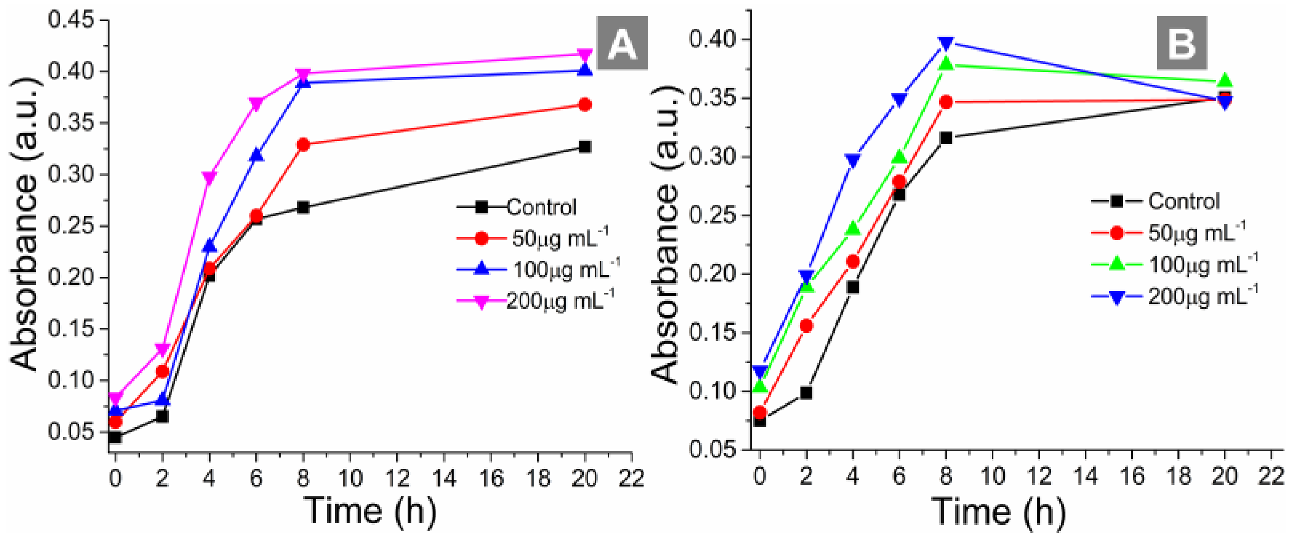

Figure S6. Growth curves of (**A**) *S. aureus* and (**B**) *E. coli* with various concentrations of the Fe_2O_3 NPs.

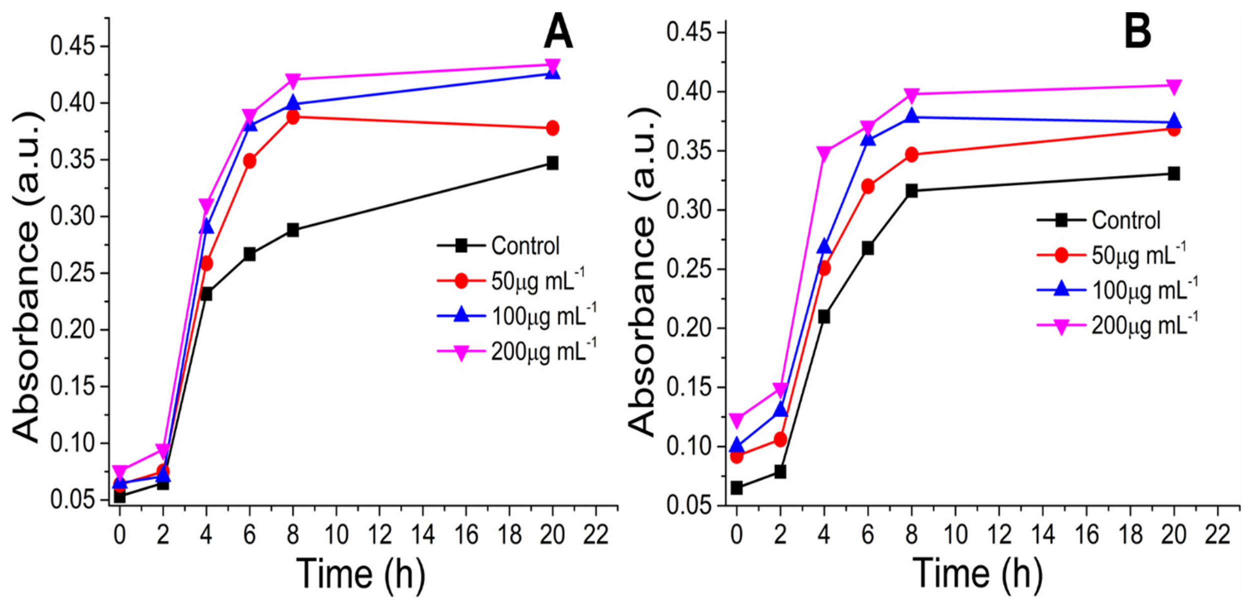

Figure S7. Growth curves of (**A**) *S. aureus* and (**B**) *E. coli* with various concentrations of the $BSiO_2$-Fe_2O_3 NPs.

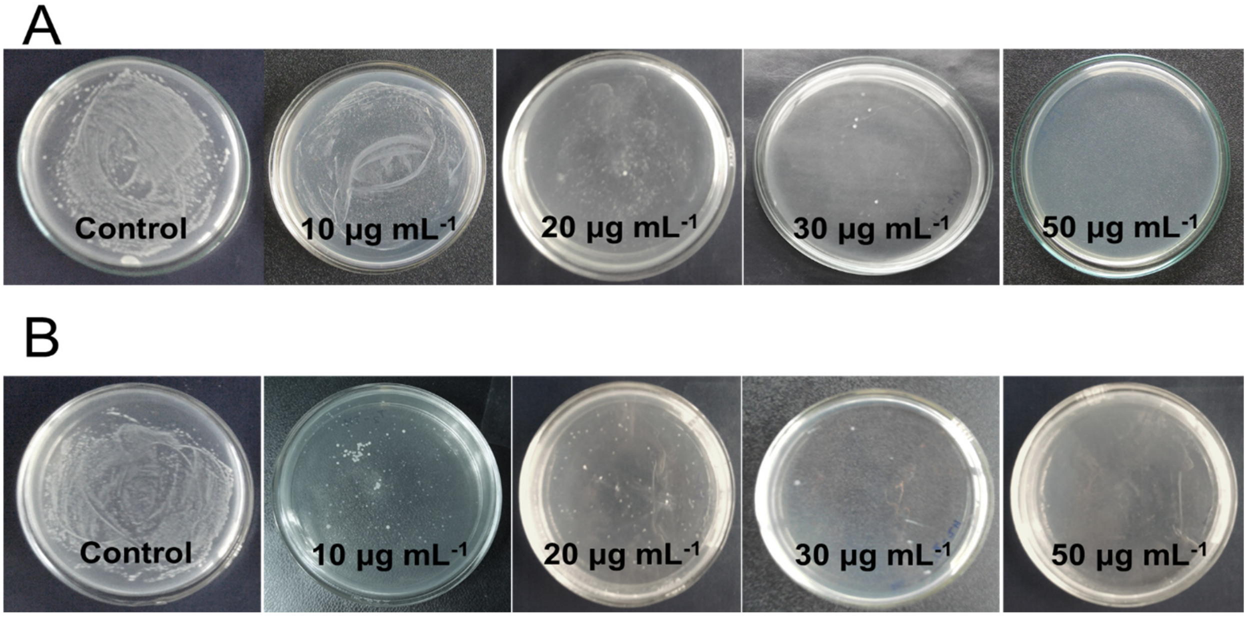

Figure S8. Growth plate photographs of (**A**) *S. aureus* and (**B**) *E. coli* with various concentrations of streptomycine antibiotics.

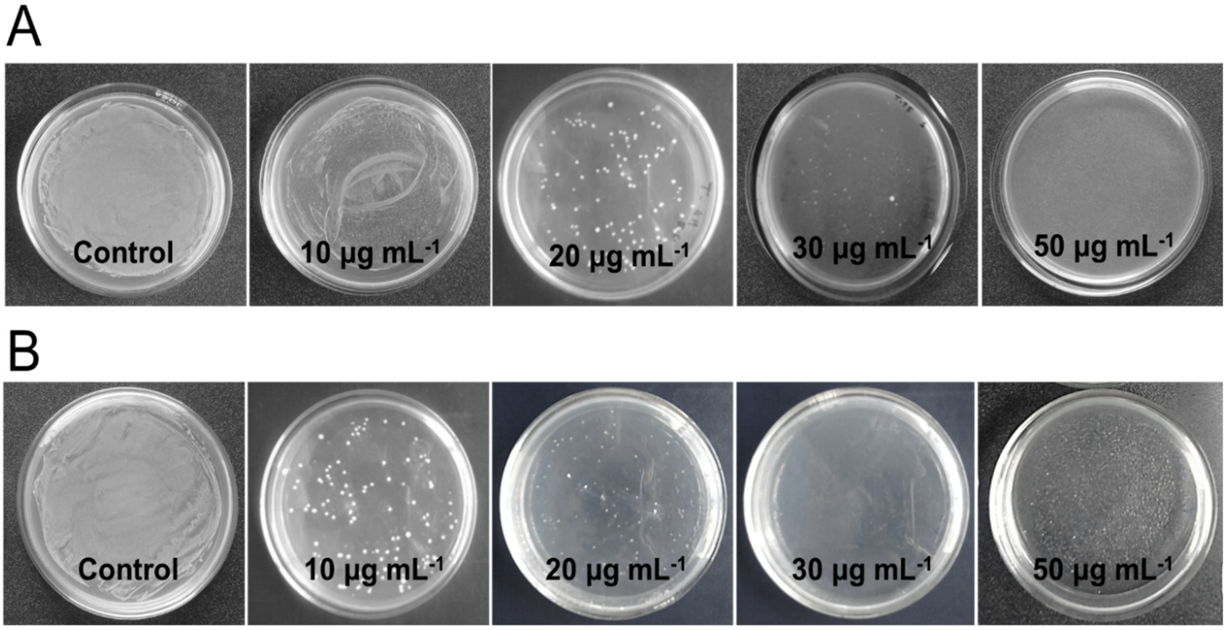

Figure S9. Growth plate photographs of (**A**) *S. aureus* and (**B**) *E. coli* with various concentrations of Ag NPs.

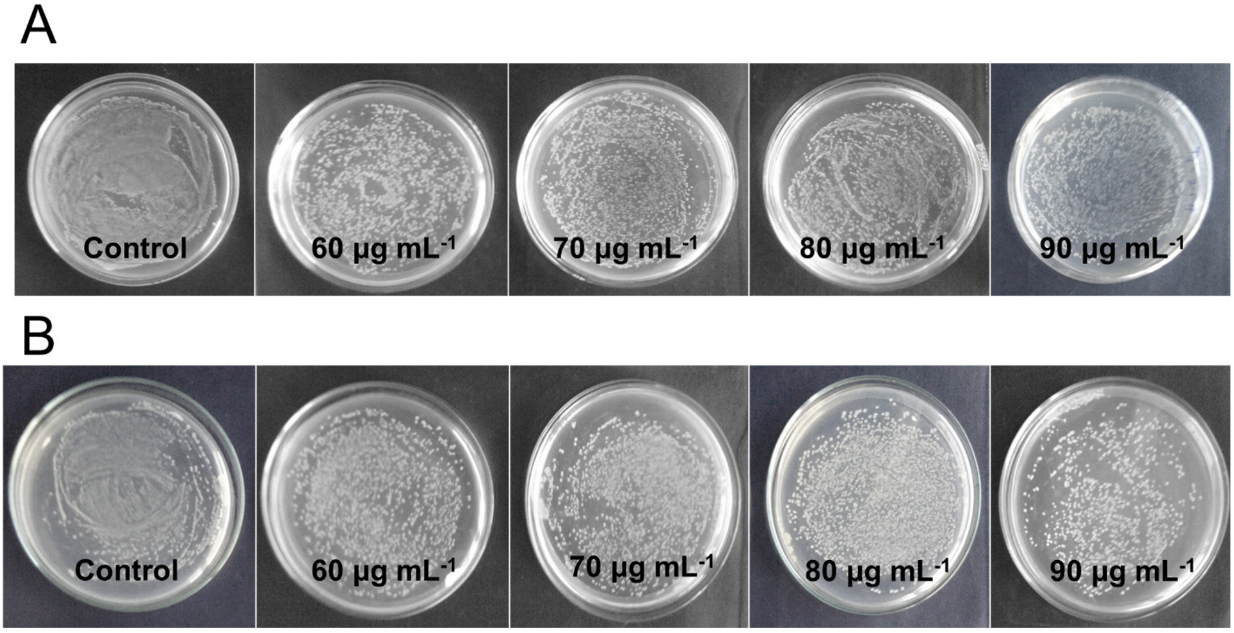

Figure S10. Growth plate photographs of (A) *S. aureus* and (B) *E. coli* with various concentrations of Fe_2O_3 NPs.

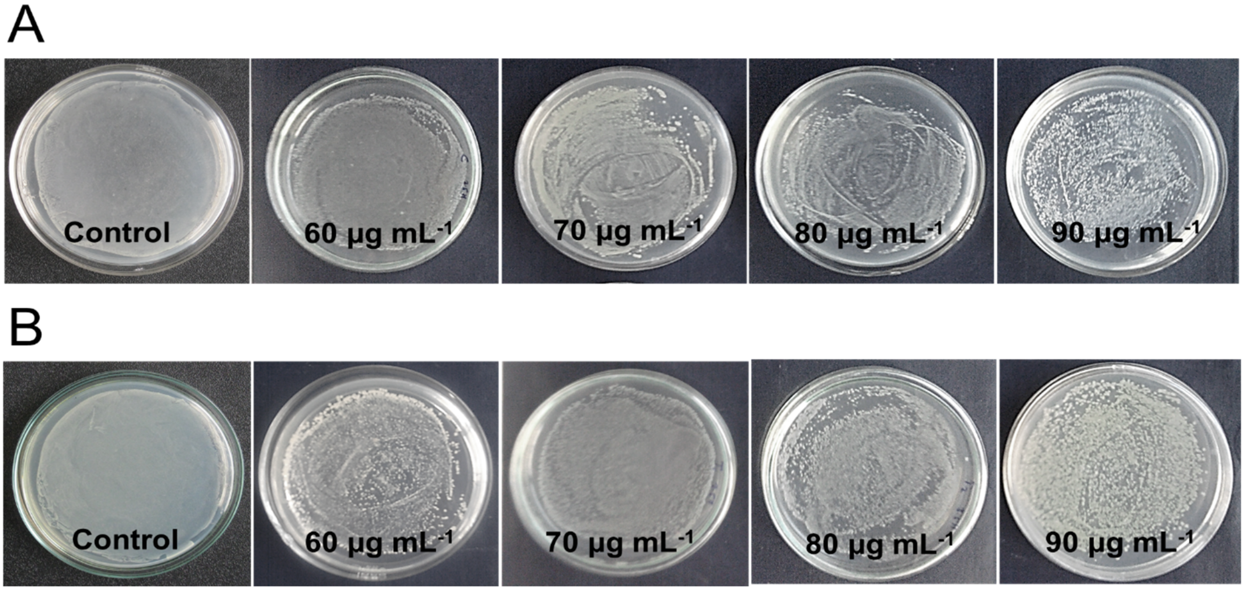

Figure S11. Growth plate photographs of (**A**) *S. aureus* and (**B**) *E. coli* with various concentrations of $BSiO_2$-Fe_2O_3 NPs.

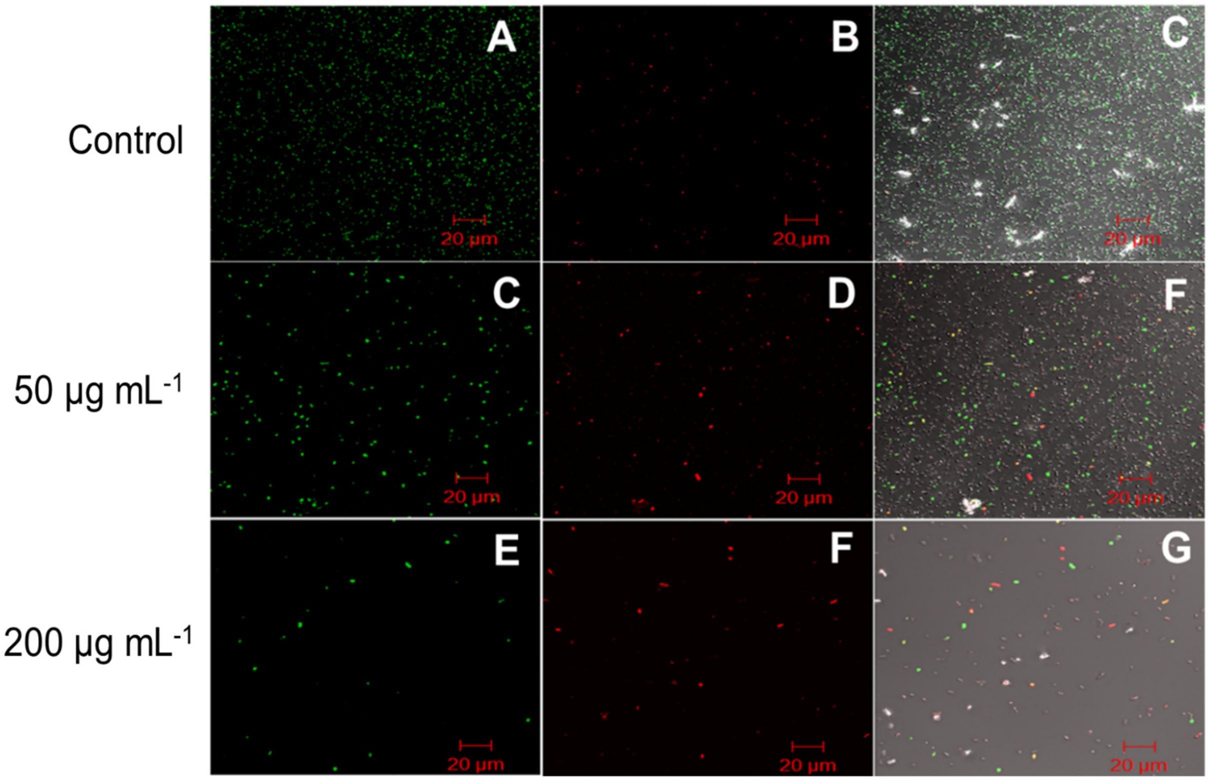

Figure S12. CLSM microscopy images of the antimicrobial activity of Ag-$BSiO_2$-Fe_3O_4 nanoparticles against *E. coli*.

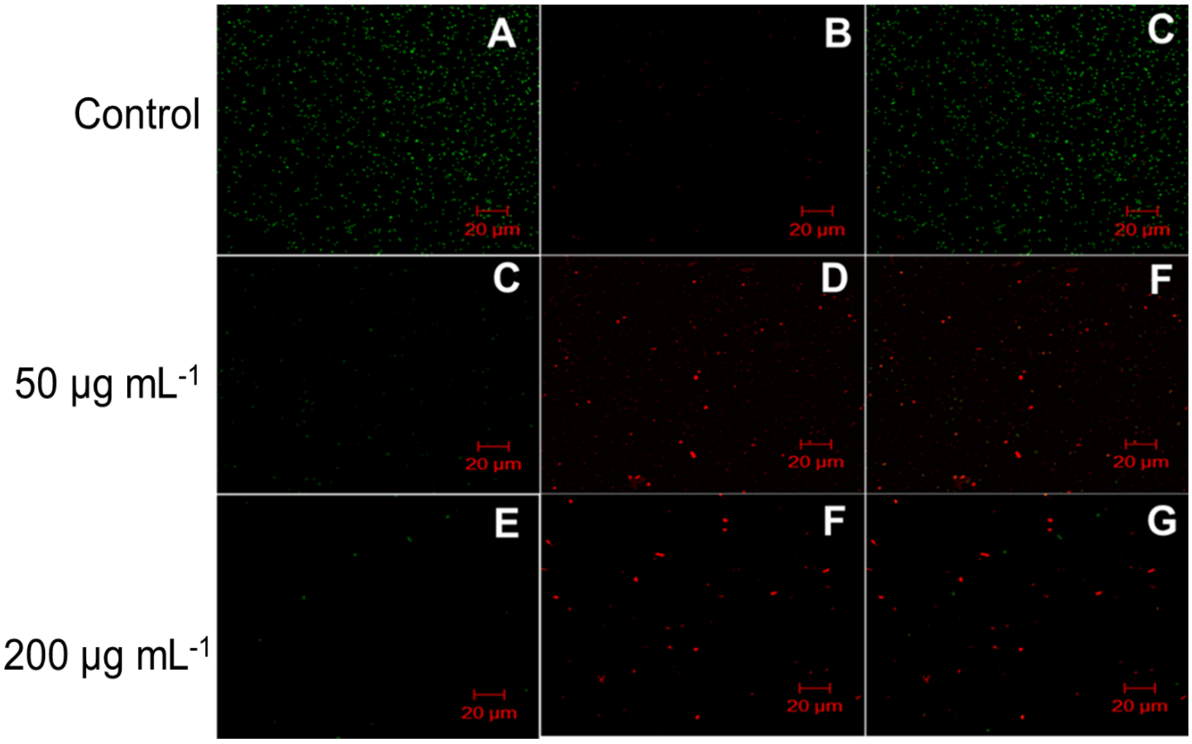

Figure S13. CLSM microscopy images of the antimicrobial activity of Ag-BSiO$_2$-Fe$_3$O$_4$ nanoparticles against *S. aureus*.
